# Nutraceuticals for Peripheral Vestibular Pathology: Properties, Usefulness, Future Perspectives and Medico-Legal Aspects

**DOI:** 10.3390/nu13103646

**Published:** 2021-10-18

**Authors:** Giuseppe Chiarella, Gianmarco Marcianò, Pasquale Viola, Caterina Palleria, Davide Pisani, Vincenzo Rania, Alessandro Casarella, Alessia Astorina, Alfonso Scarpa, Massimiliano Esposito, Monica Salerno, Nunzio Di Nunno, Matteo Bolcato, Amalia Piscopo, Erika Cione, Giovambattista De Sarro, Giulio Di Mizio, Luca Gallelli

**Affiliations:** 1 Unit of Audiology, Department of Experimental and Clinical Medicine, Regional Centre of Cochlear Implants and ENT Diseases, Magna Graecia University, 88100 Catanzaro, Italy; chiarella@unicz.it (G.C.); pasqualeviola@unicz.it (P.V.); davidepisani@gmail.com (D.P.); alessiaastorina7@gmail.com (A.A.); 2 Operative Unit of Pharmacology and Pharmacovigilance, “Mater Domini” Hospital, Department of Health Science, University Magna Graecia, 88100 Catanzaro, Italy; gianmarco.marciano@libero.it (G.M.); palleria@unicz.it (C.P.); raniavincenzo1@gmail.com (V.R.); al.cas1993@gmail.com (A.C.); desarro@unicz.it (G.D.S.); gallelli@unicz.it (L.G.); 3 Department of Medicine and Surgery, University of Salerno, 84081 Baronissi, Italy; alfonsoscarpa@yahoo.it; 4 Department of Medical, Surgical and Advanced Technologies “G.F. Ingrassia”, University of Catania, 95121 Catania, Italy; massimiliano.esposito91@gmail.com (M.E.); monica.salerno@unict.it (M.S.); 5 Department of History, Society and Studies on Humanity, University of Salento, 73100 Lecce, Italy; nunzio.dinunno@unisalento.it; 6 Legal Medicine, University of Padua, 35121 Padova, Italy; matteobolcato@gmail.com; 7 Forensic Medicine, Department of Law, Magna Graecia University of Catanzaro, 88100 Catanzaro, Italy; amaliapiscopo@gmail.com; 8 Department of Pharmacy, Health and Nutritional Sciences, Department of Excellence 2018-2022, University of Calabria, 87036 Rende, Italy; erika.cione@unical.it; 9 Research Center FAS@UMG, Department of Health Science, University Magna Graecia, 88100 Catanzaro, Italy

**Keywords:** nutraceuticals, vestibular, dizziness, vertigo, treatment, patient’s safety, clinical risk management

## Abstract

Vestibular disorders may generate complex signs and symptoms, which may alter patients’ balance and the quality of life. Dizziness and vertigo can strongly affect daily activities and relations. Despite the presence of conventional drugs, maneuvers, and surgery, another interesting therapeutic opportunity is offered by nutraceuticals. These molecules are often used in the treatment of dizziness and vertigo, but the rationale of their application is not always solidly demonstrated by the scientific evidence. Several substances have shown a variable level of efficacy/usefulness in this field, but there is lack of important evidence for most of them. From a medico-legal point of view, specific information must be provided to the patient regarding the efficacy and possibilities that the use of these preparations can allow. Administering the right nutraceutical to the proper patient is a fundamental clinical skill. Integrating conventional drug treatment with nutraceutical administration seems to be easy, but it may be difficult considering the (in part unexplored) pharmacodynamics and pharmacokinetics of nutraceuticals. The aim of the scientific community should be to elevate nutraceuticals to the same law and technical dignity of conventional drugs.

## 1. Introduction

The term “nutraceuticals” is the object of continuous debate, and to date they are described as “a food or part of a food, such as a dietary supplement, that has a medical or health benefit, including the prevention and treatment of disease” [1]. Even if these compounds are commonly used in several clinical manifestations [2,3,4,5,6], e.g., when treating vestibular diseases, their role has not been well described. Vestibular disorders include different clinical manifestations related to an alteration of the balance of visual, auditory-vestibular, and somatosensory impulses [7] with connections to cognitive and psychological areas. Vertigo affects approximately 20–30% of the general population [8]. The most common causes of peripheral vertigo/dizziness are benign paroxysmal positional vertigo (BPPV), vestibular neuritis (or acute unilateral peripheral vestibulopathy), Ménière’s disease (MD), and bilateral vestibulopathy (BV) [7,9] (Table 1).

The most common drugs employed are diuretics, betahistine (a mild H1 agonist and strong H3 antagonist, that reduces both endolymphatic pressure and vestibular nuclei activity), and antiemetic or vestibular suppressants (benzodiazepines, anticholinergics, antihistamines, phenothiazines, and ondansetron) [7,8,9,17]. Surgery embraces different choices, depending on the gravity of the illness: intratympanic steroid perfusion, ablative techniques, endolymphatic sac decompression, intratympanic gentamycin perfusion, vestibular neurectomy, and labyrinthectomy [7,9,17,20,21,22,23]. Another potential treatment strategy is intravenous glycerol [24].

Vestibular neuritis (acute unilateral peripheral vestibulopathy) is characterized by nystagmus, posture alteration, or gait abnormalities (Table 1). Vestibular suppressants and antiemetics are very useful, whereas steroids, despite the absence of solid evidence, can have a certain effect [7,9,10].

Benign paroxysmal positional vertigo affects the inner ear and causes frequent and sudden attacks of positional vertigo (Table 1). Risk factors include advanced age, ear and dental surgery, vitamin D deficiency, menopause, and vascular disorders [11,12,13,14]. The main role is played by the otoconia, which may enter the semicircular canal and change position with head movements (canalithiasis theory) or adhere to the cupula of the canal and displace it (cupololithiasis theory) [9]. The main treatment is vestibular maneuvers, performed to reposition the otoconia outside of the semicircular canals. Vestibular suppressants should not be routinely used, even if some patients may benefit from it [7,9,15,16].

Bilateral vestibulopathy is characterized by postural imbalance and unsteadiness of gait, both of which worsen in darkness and on uneven ground. Head or body movement-induced oscillopsia is also present in some patients, in particular during walking, which engenders head movements with high frequency spectral content, particularly during each heel strike (Table 1) [7,25,26].

As described in Table 1, conventional drugs, maneuvers, and surgery, are used to treat these clinical conditions. However, an interesting therapeutic opportunity can be offered by nutraceuticals. Therefore, in this manuscript we performed a narrative review on both the efficacy and safety of nutraceuticals in the treatment of peripheral vertigo.

## 2. Search Strategy

References were identified through a literature search on PubMed and Google Scholar, and through a manual search of the reference lists of identified articles. The searches combined a range of key terms (“nutraceuticals” OR “supplements” AND “dizziness” OR “vertigo” AND “vestibular”).

Duplicate manuscripts were removed after exporting references to the Mendeley reference management software (https://www.mendeley.com, accessed on 10 September 2021).

The literature search was carried out from inception to July 2021 without language restrictions, and non-English publications were translated prior to data extraction.

## 3. Main Nutraceuticals

### 3.1. Ginkgo Biloba

Extracts from *Ginkgo biloba* (EGb 761 and LI 1370 [27]) have been shown to have several effects (Table 2) which explain its role in the treatment of vertigo [27,28]. Yabe et al. [29], in an experimental model, documented the effects of *Ginkgo biloba* on the vestibular system. In a multicenter clinical trial, Sokolova et al. [30], evaluating the effects of a 12-week treatment with EGb 761 (240 mg per day) or betahistine (32 mg per day) in 116 patients with vertigo, documented that both compounds are able to induce a clinical improvement of vertigo, but that EGb 761 induced lower adverse drug reactions (70% in EGb group vs. 79% in the betahistine group) [30].

The treatment with *Ginkgo* must be avoided in pregnancy and lactation, and with children below 12 years [31], while its use should be well evaluated in poly-treated patients.

In fact, EGb 761 inhibits cytochrome P450(CYP)1A2, CYP2C9, CYP2E1, CYP3A4, and P-glycoprotein (P-gp) [32] (Table 2 and Table 3), even if the clinical role of these interaction has not been well demonstrated [33].

In contrast, the presence of flavonoids in *Ginkgo biloba* potentiates the effects of anticoagulant and antiplatelet compounds (pharmacodynamic interaction), causing a prolongation of bleeding time [34].

**Table 2:** Mechanism of action and adverse events of the main nutraceuticals/supplements used in peripheral vestibular pathology.

	Mechanism(s) of Action	Use in Peripheral Vestibular Vertigo/Dizziness	Possible Adverse Events
Alpha-lipoic acid	Cofactor in enzymatic processes that produce energy.	Non-specific.	Generally safe, even if skin and gastrointestinal disorders are described [35].
Carnosine	Antioxidant, neuromodulatory, antinflammatory, neuroprotective, stimulation of mitochondria [36].	Non-specific.	NA; probably none [37].
Citicoline	Neuroprotective, neuromodulatory, effect on phospholipid synthesis, decrease of their peroxidation, increase of blood flow, cerebral oedema reduction, increase of superoxide-dismutase activity, enhancement of acetylcholine, dopamine and noradrenalin synaptic levels, activation of SIRT-1 and neuronal repair [38,39,40].	Non-specific, but showed utility in vertigo/dizziness [38,41].	Safety comparable to placebo. Anxiety, leg oedema (more frequent), depression, falls and incontinence. Reported (not significant): stomach distress, headache, rash, cardiac abnormalities, insomnia, decrease in systolic blood pressure, excitability, restlessness, dizziness. Experimental rats models: creatinine increase, brown urine and lower urine volume in males, mineralisation in both males and females [42,43,44,45].
Coenzyme Q10	Antioxidant, ATP production [46,47].	MD-like syndromes, preventing hypoxia and improving patients’ symptoms, especially if there is a deficiency [46].	Generally safe [46].
Curcumin	Antioxidant [48].	Non-specific.	Generally safe [49,50].
Ginger	Antithrombotic, antiemetic (blocking 5HT3 receptor), antioxidant, anti-inflammatory (inhibition of COX2, lipoxygenase, and gene encoding inflammatory molecules), anti-infectious, antineoplastic, hypolipidaemic and hypoglycaemic, prokinetic, cardiovascular, thermogenic, analgesic, anti-allergic activity [51,52].	Non-specific. Effective against nausea and vomiting and with motion sickness. Controversial in vertigo/dizziness [51,53,54].	Possible adverse events mainly include gastrointestinal symptoms. Sleepiness is also documented, while allergic reactions are rare. There is no solid evidence for interactions with anti-coagulant drugs. Dizziness was described [51,52,55].
Ginkgo biloba	Neuroprotective, antioxidative, improvement of cerebral perfusion, stabilization of mitochondria, rheological properties, antinflammatory, antithrombotic and vasorelaxant action, catecholamines modulation. Activity on vestibular system (animal models) [27,28,29].	Non-specific. Generally useful in vertigo/dizziness [27].	Increase in blood pressure, dizziness, breathing rate. Poisoning: seizures, legsparalysis, unconsciousness, vomiting (susceptible subjects) [31].
Hawthorn	Acts on cardiovascular system and contributes also to relaxation and mental well-being. Hypolipidaemic, hypotensive, cardiotonic, antiarrhythmic, antioxidative activity [56,57].	Non-specific.	Generally safe. Possible adverse events: gastrointestinal symptoms, dizziness, cardiac complaints [58].
Lactium	Calming and sleep-stimulating activity [59].	Non-specific.	Generally safe. Reported: infections, gastrointestinal symptoms [59].
Lemon balm	Action on GABA system, neurocognitive effects and seems to act on cholinergic receptors. Improvements in mood and cognitive performance, antioxidant, anti-inflammatory, anti-nociceptive, hypoglycaemic, hyperlipidaemic, cardiologic, cytotoxic, antimicrobic, antispasmodic, antiepileptic effects are described. May be used in the management of various neurological pathologies or symptoms [60,61,62,63].	Non specific and generally used in vertigo/dizziness. A formulation containing lemon balm was effective in BPPV [61].	Generally safe [62]. Rare adverse events (similar to placebo): thyroid hormone inhibition, dizziness, nausea, vomiting, palpitation, wheezing, agitation, increased appetite, EEG changes, reduced alertness, increased intraocular pressure [60]. In mice: prone position, decreased motor activity, difficult breathing, tremors (which resolved spontaneously) [62].
Magnesium	Important enzymatic cofactor. Pplays a role in the prevention of various pathologies. Involved in the structural function of nucleic acids, mitochondria, proteins, transport of other ions, DNA/RNA synthesis and aerobic/anaerobic energy production, and many other functions. It can reduce catecholamines [64,65].	Its deficiency is associated with vertigo [64,66]. Evidence in the treatment of headache/migraine concomitant to vertigo/dizziness, ISHL with vertigo/dizziness, post-stapedectomy vertigo [9,64,67,68,69].	Hypermagnesemia: vomiting, nausea, headache; absent tendon reflexes, hypotension, somnolence; cardiovascular alteration and hypoventilation; cardio-respiratory arrest, coma, death [64,70].
Omega-3 fatty acids	Important role in the production of eicosanoids, such as prostaglandins and leukotrienes. They improve cardiac filling, myocardial efficiency, anti-inflammatory effects, vasodilation. They also regulate ion channel function and provide cellular membrane stability, since they are incorporated in membrane phospholipids [71,72].	Possible future option in MD [71].	Generally safe. Possible adverse events: skin eruptions and gastrointestinal symptoms were the most frequent, with the possibility of some laboratory parameter alteration. In dogs and cats: altered platelet and immune function [73,74].
Orthosiphon	Antioxidant, anti-inflammatory, analgesic, antihypertensive, renal and hepatoprotective, diuretic, gastroprotective, and many other properties [75].	Non-specific.	Considered safe. Liver hypertrophy is a possible adverse event [75].
Polygonum	Immunomodulating, antioxidant, anti-cancer, neuroprotective, anti-ageing, hepatoprotective, anti-hyperlipidaemia, anti-inflammatory [76].	Non-specific.	Generally safe. Certain components or preparations may generate nephrotoxicity, hepatotoxicity, lung damage [76].
Sage	Anti-inflammatory, antidepressant, anxiolytic, antioxidative, antimicrobial, anticancer, antinociceptive, antidementia, hypoglycaemic, hypolipidaemic properties. It inhibits AChE (cholinergic activity) and inhibits the GABA_A_ receptor (through thujone) [77,78,79,80].	Non-specific. Effective in vertigo/dizziness [79].	Generally safe. Reported signs and symptoms include: salivation, gastrointestinal symptoms, tachycardia, skin eruption, hot flushes, hypersensitivity, cyanosis, increased blood pressure, and convulsion [77,79,80].
SPC-flakes *	Contain AF. It may regulate ions and water, interacting with aquaporins and modulating chloride homeostasis [81].	Used in MD [81,82].	No adverse events reported [81,83].
Vinitrox	Combination of apple and grape polyphenols with vasodilator and antioxidant effect [61].	Non-specific.	NA.
Vitamin B	Vitamin B6 protects circulation and seems to facilitate vestibular system, acting on vertigo [61,84,85]. Vitamin B2, B3, and B6 aid nervous system. B2 and B3 also maintain mucous membranes [56].	Vitamin B deficiency, in general, may lead to neurological symptoms, also dizziness and vertigo [86,87].	Possible toxicity (rare). Vitamin B12: transientchromaturia, skin eruptions, CNS manifestations, increased bloodpressure, gastrointestinal symptoms [88]. Vitamin B3: flushing, skin and gastrointestinal manifestations, headache, light-headedness [89]. Vitamin B6: neurological symptoms [90].
Vitamin C	It is a radical scavenger that gave an improvement in MD control, considering an oxidative insult as a basis for MD origin: controversial in a study. Vitamin C also contributes to nervous system function [91,92,93].	Non-specific. Maybe useful in MD [92].	Rare adverse events: gastrointestinal symptoms, kidney stones in men [94].
Vitamin D	Vitamin D deficiency/alterations of calcium metabolism are also risk factors for recurrence, and probably pathogenic factors of BPVV, immunomodulatory activity (immune system role on MD) [95,96,97,98].	Vitamin D supplementation in BPVV showed a 24% lower recurrence for patients with low serum levels. Class III evidence. Possible role in MD [95,96,97,98].	Gastrointestinal symptoms. Intoxication: hypercalcemia, hypercalciuria. This potentially leads to muscle weakness, hypertension, neuropsychiatricdisturbances, gastrointestinal upset, polyuria and polydipsia, renal calculi, and, in extreme cases, renal failure, arrhythmias [95,99].
Vitamin E	Antioxidant [47,100].	Non-specific.	Intoxication: increase of bleeding risk, hepatobiliary disfunction, malabsorption of other fat-soluble vitamins [101].
Zinc and copper	Antioxidant [56].	Non-specific.	Zinc (intoxication): Gastrointestinal symptoms, muscle cramps, rare cases of kidney injury [102]. Copper (intoxication): neurological, liver damage (similar to Wilson’s disease) [103,104].

* They do not fit the definition of nutraceutical or supplement, and are more like an enriched food. Abbreviations: AChE, acetylcholinesterase; AF, antisecretory factor; ATP, adenosine triphosphate; BPPV, benign paroxysmal positional vertigo; CNS, central nervous system; COX, cyclooxygenase; DNA, deoxyribonucleic acid; EEG, electroencephalogram; GABA, gamma-Aminobutyric acid; ISHL, idiopathic sudden hearing loss; MD, Ménière’s disease; NA, not available; RNA, ribonucleic acid; SIRT, sirtuin; 5HT, 5hydroxytryptamine.

**Table 3:** Pharmacokinetic and pharmacodynamic interactions of nutraceuticals supplements used in the treatment of peripheral vestibular vertigo/dizziness.

	Metabolism by CYP or Transporters	Action on CYP or Transporters	Theorical or Factual Pharmacodynamic Interactions
Citicoline	NA.	NA.	Increases vertigo/dizziness feeling: interacting with antibiotics (aminoglycosides, macrolides, glycopeptides), antimalarial drugs, NSAIDs, and acetylsalicylic acid, which also cause vertigo/dizziness [45,105]. Increased activity of antihypertensive drugs [42]. Increased levels of acetylcholine [38,39]. Increased levels of dopamine, noradrenaline and serotonin; this generates interference with drugs acting on these pathways [38,39].
Ginger	NA.	In vitro inhibition of CYP2C9, 2C19 2D6, and CYP 3A4 [51,106]. Inhibition of P-gp in vitro [51,106].	Anti-allergic activity: increased effects of antihistamines. Increased risk of bleeding [51,52]. Hypoglycemic effects [51]. It blocks 5HT3 receptor similarly to ondansetron; increased activity [51]. Anti-allergic activity: increased effects of antihistamines with increased risk of sleepiness [51,52].
*Ginkgo biloba*	NA.	Inhibition of CYP1A2, CYP2C9, CYP2E1, CYP3A4, and P-gp [32].	Increased effects of cilostazol or anticoagulants with increase in bleeding time [31,34]. Increase in vertigo/dizziness feeling: interaction with antibiotics (aminoglycosides, macrolides, glycopeptides), antimalarial drugs, NSAIDs, and acetylsalicylic acid, which causes vertigo/dizziness [31,105]. Proconvulsant effect: interaction with anti-epileptic drugs [107]. Increase of acetylcholine levels [108]. Increase of dopamine and noradrenaline levels [108].
Hawthorn	No strong information. Some authors described a new CYP450 enzyme responsible for terpenoids C-2α hydroxylation [109].	NA.	Increased activity of beta-blockers, digitalis, and hypotensive drugs [57]. Increased activity of antihypertensive drugs [57].
Lemon balm (*Melissa officinalis*)	NA.	NA.	Action on GABA receptors: may increase the effects of BDZ [61,63]; Hypoglycemic effects [60]. Increase of vertigo/dizziness feeling: interaction with antibiotics (aminoglycosides, macrolides, glycopeptides), antimalarial drugs, NSAIDs, and acetylsalicylic acid, which causes vertigo/dizziness [60,105]. Increase in intraocular pressure: pay attention to patients with glaucoma [60]. Action on acetylcholine pathways: may interact with cholinergic and anticholinergic drugs [63]. Associated with tremors: pay attention to patients with Parkinson’s disease [62].
Magnesium	NA.	NA.	Increased activity of antihypertensive drugs. Loop diuretics may cause hypomagnesemia [110].
Omega-3 fatty acids	Omega-3 and omega-6 can be metabolized by CYP1A1, CYP2E1, CYP2C, CYP2J2, CYP4A, CYP4F, and other isoforms as efficient alternative substrates of arachidonic acid metabolizing CYP enzymes [111].	NA.	NA.
Ortosiphon	NA.	In vitro inhibition of CYP2C19, CYP2D6, and CYP3A4 [112,113].	Anti-seizure activity: may increase anti-epileptics activity [114]. Increased activity of antihypertensive drugs [75].
Polygonum	NA.	Induction of CYP2C9 and CYP3A4 [115]. Inhibition of CYP3A and MRP [116].	May be useful in Parkinson’s disease [76].
Sage (*Salvia officinalis*)	NA.	Components act on CYP450: among phenolic acids, TSIIA inhibited CYP2C9/3A4, whereas SAB induces it [117]; among the terpenoids, salvinorin A showed to be a CYP1A1 2C18, 2D6, and 2E1 substrate [117]. Inhibition of CYP1A2, CYP2C9, CYP2C19, CYP2D6, CYP2E1, CYP3A4 [117]; Induction of CYP1A2 and CYP3A4 [117]. Some compounds showed activity on P-gp and OAT [117].	Inhibition of GABA_A_ receptors: decreased effects of BDZ [77,80]; Inhibition of AChE: this may lead to an interaction with cholinergic or anticholinergic drugs [77]. Increase risk of bleeding [117]. Hypoglycemic effects [79,117]. Increase of vertigo/dizziness feeling: interaction with antibiotics (aminoglycosides, macrolides, glycopeptides), antimalarial drugs, NSAIDs, and acetylsalicylic acid, which causes vertigo/dizziness [105,117]. Proconvulsant effects: interaction with anti-epileptic drugs [79,80]. It may reduce tremors: enforces the action of drugs used in neurological diseases [79]. Associated with both increased and decreased blood pressure [77,80].

Abbreviations: AChE, acetylcholinesterase; BDZ, benzodiazepines; CYP, cytochrome P 450; GABA, gamma-Aminobutyric acid; MRP, multidrug resistance protein; NA, not available; NSAID, nonsteroidal anti-inflammatory drugs; OAT, organic anion transporter; P-gp, p-glycoprotein; SAB, salvianolic acid B; TSIIA, tanshinone IIA.

### 3.2. Ginger

Ginger (*Zingiber officinale)* contains several active compounds: gingerols, shogaols (phenolic compounds), and terpenes (for example, zingiberene) [52,118], that are able to induce antioxidant and anti-inflammatory effects (Table 2).

Even if some authors did not report an effect the on vestibular system [51], others described an effect in reducing vertigo/dizziness and motion sickness syndrome [51,53,54].

Ginger is also associated with dizziness [55], increasing doubts about the solidity of the evidences.

Possible adverse events mainly include gastrointestinal symptoms (reflux, bad taste, diarrhea, and abdominal discomfort) and sleepiness (Table 3). Even if the development of drug interactions in patients using anti-coagulants were reported, these would not have a solid basis of evidence [51,52].

Ginger inhibits CYP 3A4, 2D6, 2C9, 2C19, and P-gp in vitro, but there are insufficient data to describe the development of drug interactions in clinical settings [51,106].

### 3.3. Citicoline

Citicoline is an intermediary in the phospholipids synthesis chain, with multiple properties (Table 2 and Table 3), and which seems to have an effect on vertigo/dizziness [38,41], even if there is no strong evidence to support it [38,119]. Petrova et al. [41] documented a dose-dependent efficacy of citicoline (up to 2000 mg/day) in the management of central and ischemic vertigo. However, enrolled patients were treated with betahistine, so it is not easy to evaluate if clinical efficacy was related to citicoline, betahistine, or both. Martines et al. [38] used a combination of citicoline, ginger, B6 vitamin, lemon balm, and ViNitrox, and obtained a positive effect. In contrast, the development of dizziness was described in patients treated with citicoline at a high dosage [45].

Finally, in a previous sequential trial performed with 2298 patients with moderate-to-severe acute ischemic stroke, with patients randomly given citicoline (1000 mg every 12 h i.v. during the first 3 days and then orally, 500 mg bid, for a total of 6 weeks) or a placebo, Dávalos et al. [120] failed to report a difference in the development of adverse drug reactions or drug interactions between citicoline and the placebo (Table 3). However, the safety of citicoline must be evaluated carefully for its use in pregnancy, lactation, and childhood [42].

### 3.4. Magnesium

Magnesium is an enzymatic cofactor (Table 2) and its deficiency is associated with a lot of pathological issues (Table 2), including enclosed vertigo [64,66]. Esposito et al. administered a combination of *Griffonia simplicifolia*/magnesium as a prophylaxis to treat childhood motion sickness, and demonstrated a better outcome vs. the control group. A possible application of magnesium is revealed by the demonstration of its effectiveness in the treatment of headaches and migraines, alongside vertigo/dizziness [9,64,67]. It may also be used to treat vestibular migraines, because these patients reported lower magnesium levels [121]. In patients with idiopathic sudden hearing loss, the treatment with magnesium reduced vertigo and vestibular damage [68]. Magnesium has also been used in post-stapedectomy vertigo, alongside vitamin B2 and conventional migraine medication [69].

### 3.5. Lemon Balm

There are little data concerning the use of lemon balm *(Melissa officinalis)* in the treatment of vertigo/dizziness [60]. The formulation Vertigoval^®^ (Valeas, Italy), which contains ginger, lemon balm, ViNitrox, vitamin B6, and citicoline, showed an improvement of residual dizziness in VPPB compared with a placebo, and this effect is probably related to the action of lemon balm on the GABA system [61] (Table 3). The action on the GABA system produces an anxiolytic effect, similar to benzodiazepines [63]. It is interesting to state that benzodiazepines are also used in the management of vertigo/dizziness [17], and this increases the importance of lemon balm. There are no data regarding the safety of this compound, but in an experimental study, the administration of oregano and lemon balm induced the prone position, decreased motor activity, created difficulty in breathing, and induced tremors in mice. Human studies showed rare adverse events (not significantly different from the placebo) [60], but it should be used carefully in pregnancy, lactation, or childhood [62].

### 3.6. Sage

Sage (*Salvia spp.*, above all *Salvia officinalis)* is a plant with important medical properties due to its metabolites and components [77,78,79] (Table 2). *Salvia* does not usually generate severe adverse events [77,79,80] (Table 3). Some constituents in *Salvia* act on CYP450: for example, among phenolic acids, tanshinone IIA (TSIIA) inhibited CYP2C9/3A4, whereas salvianolic acid B (SAB) induced it; among the terpenoids, salvinorin A is a CYP2D6, 1A1, 2C18, and 2E1 substrate. CYP3A4, CYP1A2, CYP2E1, CYP2D6, CYP2C9, and CYP2C19 were inhibited by some molecules, while CYP3A4 and CYP1A2 were induced by others. Some compounds showed activity on P-gp and organic anion transporters (OATs), but all these data were ambiguous and not clear enough to determine the in vivo activity of the whole medicinal use of *Salvia* [117].

### 3.7. Omega-3 Fatty Acids

Omega-3 polyunsaturated fatty acids include stearidonic acid, docosapentaenoic acid, docosahexaenoic acid, eicosapentaenoic acid, and α-linolenic acid [122]. They perform multiple healthy actions on the human body: they are helpful in treating Alzheimer’s disease, cancer, cardiovascular disease, and the development of diabetes and neurological conditions [123]. Omega-3 fatty acids could represent a potential future option in the treatment of Meniere’s disease because they are able to induce hemodynamic changes (Table 2).

Omega-3 fatty acids are well tolerated, even if their uses are related to the development of adverse drug reactions (Table 3).

### 3.8. Specially Processed Cereals (SPC) Flakes

Antisecretory factor (AF) is a protein produced in the pituitary gland which fights infections and which performs an immunity role [81] (Table 2). Moreover, it is able to regulate ions and water, interacting with aquaporins and modulating chloride homeostasis (Table 2). Therefore, it can be used in patients with Meniere’s disease. Viola et al. documented an improvement in tinnitus and vertigo in patients treated with SPC vs. intravenous glycerol and dexamethasone [81]. In contrast, Scarpa et al. [82] failed to report a significant difference between glycerol, dexamethasone, and SPC, even if the time of the follow-up was lower (12 months, compared to 24 months in Viola’s study). Similarly, Ingvardsen and Klokker [124] showed no difference in the SPC flakes group compared with the placebo group, even if the follow up period was shorter.

These data support the idea that a long period of treatment is necessary to obtain clinical efficacy during the treatment with SPC flakes. However, no adverse drug reactions have been described.

### 3.9. Other Compounds/Supplements

Vertigoval^®^ (Valeas, Italy) is a formulation composed of ginger, ViNitrox, citicoline, vitamin B6, and *Melissa officinalis*. ViNitrox is a combination of apple and grape polyphenols with vasodilator, which has an antioxidant effect. Vitamin B6 protects circulation and seems to facilitate the vestibular system [61]. The effects of vitamin B6 on vertigo have been known for decades [84,85]. Vertigoval^®^ showed an improvement of symptoms and instability in patients [61].

Vestanol^®^ (Erbozeta, Chiesanuova, Republic of San Marino) is a product which contains Ginkgoselect^®^ plus Phytosome^®^, hawthorn, ginger, orthosiphon, polygonum, B vitamin complex, zinc, and copper. The most important active substances are terpenes, flavonoids, and oligomeric proanthocyanidins [57]. It can be used to treat gastrointestinal symptoms [125].

Zinc and copper have antioxidant effects, whereas vitamin B2, B3, and B6 aid the nervous system. B2 and B3 also maintain mucous membranes [56]. Vitamin B deficiency may lead to neurological symptoms, as well as dizziness and vertigo [86,87]. No correlation between vitamin B12, homocysteine, folic acid levels, and vertigo has been found [126], although were reported cases of patients affected by vestibular pathology of vascular origin, with homocysteine increases resulting in Meniere symptoms [127,128].

Vertiwin^®^ (Pharmawin, Milan, Italy) is based on citicoline, ginger, Ginkgo select Phytosome, magnesium, vitamin C, and vitamin B complex [91]. Vitamin C and other radical scavengers led to an improvement in Meniere’s disease. A study evaluated this effect, but enrolled few patients, was not placebo controlled, and was biased by the fact that some patients would recover spontaneously, even without radical scavenger administration [92]. Vertase^®^ (Polifarma S.p.A, Roma, Italy) contains *Melissa*, ginger, citicoline, and bioflavonoids from fruit.

Vertistop^®^ L and D (Difass, Cerasolo, Rimini, Italy) are two different formulations. Vertistop^®^ L is made by LICA^®^ (alpha-lipoic acid, carnosine, and zinc) and curcumin [48]. Carnosine [36], alpha-lipoic acid [129], and curcumin have antioxidant effects (Table 2) [48].

Vertistop^®^ D is formed by LICA^®^, vitamin D, and vitamin B complex. Vitamin D works on calcium metabolism but has been studied also in vertigo/dizziness. In a non-blinded, non-placebo-controlled study, vitamin D supplementation in BPVV showed a 24% reduction in the recurrence of symptoms [95,96]. Another study provided class III evidence for its administration in this group of patients [97]. Other authors suggest that vitamin D might play a role in treating Meniere’s disease, putting emphasis on the immunomodulatory activity of this compound [98].

Acuval^®^ Vert (Sharper, Milan, Italy) is composed of coline, ginger, Ginkgoselect^®^ Phytosome^®^, Qter^®^ (coenzyme Q10), vitamin B complex, vitamin E, and lactium (hydrolyzed milk proteins) [47]. Vitamin E shows antioxidant properties [47,100], lactium has calming and sleep stimulating activity [59], and coenzyme Q10 (an antioxidant and important part of ATP production) acts in Meniere’s disease-like syndromes, preventing hypoxia and improving patients’ symptoms, especially if there is a deficiency [46].

## 4. Discussion

The debate on the use of nutraceuticals in clinical practice is not easy, because their use is not always reported in guidelines or reviews [10,15,17].

Plants and fruits are an important part of this business: despite the common enthusiasm for the natural origin of the product, these formulations may hide a latent danger. Even if the broader category is the same for different forms of *Salvia* or *Melissa* or other plants, there may be subtle chemical and pharmacological differences from one species to another, and the extracts could vary strongly and create uneven effects depending on the clinician’s ability or willingness [77,117,130]. This could generate confusion, especially in customers, and lead to the creation of non-specific or inefficient products.

A crucial problem is the absence of pragmatic pharmacokinetic studies: a lot of substances are part of the plants or of their formulations, and each of them act with proper pharmacodynamic and pharmacokinetic mechanisms [32,60,79,112,130,131]. This situation creates two problems/possibilities: the knowledge of single-component pharmacokinetics is not definitively descriptive of the pharmacokinetics of the whole compound/plant; the isolation of the most curative molecule from the plant may lead to a more specific therapy, regulating the quantity and quality of the treatment.

Flavonoids (for example, quercetin), phenolic acids, terpenes, and terpenoids are often the main causal agents of the benefits of these therapies (contained variously in ginger, sage, lemon balm, orthosiphon, and hawthorn [31,57,60,75,76,79,118,130]). However, they are administrated alongside the non-useful or non-specific molecules contained in the plants, which should be removed by the pharmaceutical industry to produce a purer compound, nearer to the definition of a drug. In this sense, understanding with total certainty which chemical entity mediates the specific therapeutic action is fundamental: substances such as quercetin (present in *Melissa* and *Salvia,* for example), resveratrol (in *Polygonum*), curcumin, and epigallocatechin gallate have been studied deeply in this sense [132].

These products are generally considered safe and relatively free of interactions, but this is not always true, although it is very difficult to predict when an adverse event would happen [133].

Food or drug action on the gastrointestinal tract may affect nutraceutical and supplemental absorption. Vitamin D absorption is influenced by the species of dietary lipids (monounsaturated and long-chain fatty acids seem to increase vitamin D absorption effectiveness). Dietary fiber may affect vitamin D absorption, since they may influence micelles formation, alter emulsification and triacylglycerol lipolysis, and increase the viscosity of the chyme. All these actions will result in alterations in the release and diffusion of fat-soluble microconstituents. Inhibitors of lipid absorption, such as orlistat, may reduce the absorption of vitamin D, whereas vitamin E may be an enhancer of this process [134]. Magnesium and zinc may interfere with tetracycline antibiotic and fluoroquinolone absorption by binding to these drugs. Antacids, containing magnesium and zinc, may be enhancers of this effect [135,136]. Furthermore, meals based on yogurt, cheese, and milk contain calcium, which is another cation responsible of this reaction. Therefore, it may worsen the cited interaction [137]. In general, food and meals influence drug absorption and activity, depending on meal composition, and determine the change in viscosity of the gastrointestinal tract, pH variations, retarded gastric emptying, changes of gastrointestinal flora, visceral blood flow, and bile secretion [137]. Nevertheless, to our knowledge, there are no specific studies on nutraceuticals.

Pharmacokinetic interaction with food should be examined with more attention. Grapefruit juice is a CYP3A4 and P-gp inhibitor because of the action of its flavonoid naringenin, whereas grape juice is a CYP3A4 inductor. None of the nutraceuticals used for the treatment of peripheral vestibular vertigo is a real CYP substrate (Table 3). However, in vitro or theoretical inhibition/induction of CYP450 by these substances may add to that of grapefruit juice and grape juice. Several studies have been conducted focusing on other kinds of juice, such as cranberry, orange, and pomegranate juice [138].

Moreover, nutraceuticals are rich in terpenes, polyphenols, and other substances contained in aliments, especially fruit and vegetables. Therefore, the concomitant administration of nutraceuticals and food containing similar/different compounds may be responsible of additive or negative interactions. Berries, dark chocolate, soy, green tea extract, cereals, liquorice, dry legumes, coffee, and wine are all rich of different kind of natural chemical agents that need further studying to assess their clinical relevance. Besides this, agonistic/antagonistic action on CYP450 must be held to account. For example, green tea extract may inhibit CYP3A4, whereas ginseng inducts it [132,139].

NSAIDs, anti-hypertensive drugs, antihistamines, benzodiazepines, and some antibiotics are often obtained by patients without medical prescription and may interact with nutraceuticals/supplements. Furthermore, ginger, *Ginkgo Biloba*, *Orthosiphon*, *Polygonum*, and *Salvia* may act in various ways on CYP3A4 (e.g., some macrolides, some benzodiazepines), 2C9 (e.g., NSAIDs), 2C19 (e.g., proton pump inhibitors), 2D6 (e.g., some antidepressants), 1A2 (e.g., duloxetine, caffeine), and P-gp substrates (e.g., loperamide), and are sometimes used without medical prescription [140,141,142,143]. Although clinical relevance needs to be assessed, all these possible pharmacodynamic/pharmacokinetic interactions with nutraceuticals are summarized in Table 3.

The absence of dedicated national and international institutions, which describe/regulate some aspects of nutraceutical administration, such as the quantity, and the component of the formulations is a major issue.

New, real-life data are required to state the nature of in vivo human interactions, after the demonstration of nutraceuticals capacity to modulate several enzymes or transporters in vitro.

## 5. Medico-Legal Aspects

The theme of the administration and prescription of nutraceutical formulations reveals some peculiar aspects from the medico-legal point of view, especially about information and consent to treatment. From the point of view of international legal principles, it is now commonly agreed that before administering any substance and carrying out any surgical procedure, it is necessary to adequately inform the patient of the expected benefits and any risks of the proposed activity. Art. 3 of the Charter of Fundamental Rights of the European Union, proclaimed for the first time on 7 December 2000 in Nice, and then on 12 December 2007 in Strasbourg, states: “Right to the integrity of the person. 1. Everyone has the right to respect for his or her physical and mental integrity. In the fields of medicine and biology, the following must be respected in particular: (a) the free and informed consent of the person concerned, according to the procedures laid down by law; (b) the prohibition of eugenic practices, in particular those aiming at the selection of persons; (c) the prohibition of making the human body and its parts as such a source of financial gain; (d) the prohibition of the reproductive cloning of human beings. (…)”. Article 11 on the freedom of expression and information states: “1. Everyone has the right to freedom of expression. This right shall include freedom to hold opinions and to receive and impart information and ideas without interference by public authority and regardless of frontiers. 2. The freedom and pluralism of the media shall be respected”. Consent obtained after providing information and expressed without conditioning is therefore an essential requirement for performing activities that affect the person’s physical and mental integrity. Article 5 of The Convention on Human Rights and Biomedicine (or the Oviedo Convention), adopted on 4 April 1997 at the behest of the European Council, ratified in Italy by Law No. 145 dated 28 March 2001, states: 1. An Intervention in the health field may only be carried out after the person concerned has given free and informed consent to it. 2. This person shall beforehand be given appropriate information as to the purpose and nature of the intervention as well as on its consequences and risks. 3. The person concerned may freely withdraw consent at any time (…). Article 10 on private life and right to information, states: “(…) 2. Everyone is entitled to know any information collected about his or her health. However, the wishes of individuals not to be so informed shall be observed”. Article 5 lays down the obligation to inform, apparently portraying said obligation in a limited light, because (1) the information is presented as second to the objective of obtaining consent, and (2) its contents relate only to an intervention in the healthcare field. This provision must therefore be considered an addition to that provided for in Article 10, the contents of which, specifically regarding information as such, are wider in scope, referring to the global view of the concept of health and not to one specific intervention. Therefore, the information contained in Article 10 pertains to the person’s health in its entirety and that of Article 5 to medical intervention dependent on consent. In addition to the obvious information with the specific aim of obtaining consent, “basic” information must also be provided free from any contingent objective, which enriches the person’s cultural awareness and constitutes the method for enabling him or her to consciously define his or her life plan. The Oviedo Convention does not specify who is responsible for informing the person. However, the provision in Article 5 is related to “interventions in the healthcare field”. Therefore, it relates not only to the medical field but, more extensively, to the healthcare field. Consequently, every healthcare professional needs to inform the patient, in the context of the specific relationship that he or she has with the patient, with any information pertinent to his or her specific role and obtain consent relating both to the prescription of a certain activity (in line with a specific objective and/or project) and to its execution.

On this aspect, the Italian Law No. 219 of 22 December 2017—“Provisions for informed consent and advance treatment directives”—is an extraordinary contribution to the issue of information and consent (9–10). Article 1 states: “1. This law, in observance of the principles referred to in Articles 2, 12 and 32 of the Constitution and Articles 1, 2 and 3 of the Charter of Fundamental Rights of the European Union, protects the person’s right to life, health, dignity and self-determination and stipulates that no medical treatment shall be initiated or continued unless the person concerned has given free and informed consent, except in cases expressly provided for by law. 2. This promotes and enhances the relationship of care and trust between a patient and doctor, which is based on informed consent, where the patient’s decision-making autonomy meets the doctor’s expertise, professional autonomy and liability. The healthcare professionals who make up the medical team contribute to this care relationship, based on their respective skills. 3. Everyone has the right to know his or her health condition and to be informed in a complete and up-to-date manner that he or she understands regarding any diagnosis, prognosis, risks, and benefits of any diagnostic tests and recommended medical treatment, as well as regarding the possible alternatives and consequences of refusing or withdrawing from medical treatment and diagnostic tests.” The law confirms the patient’s right to be informed about several elements: health condition, diagnosis, prognosis, risks and benefits of diagnostic tests and recommended medical treatments, possible alternatives, and the consequences of refusing the recommended treatment. The law explicitly states that information must be given to the patient in a complete, up-to-date manner that he or she understands. Therefore, the procedure to be followed in the doctor–patient relationship should include a communicative method appropriate for the patient’s condition and capability and be focused upon his or her needs and characteristics without losing its complete and up-to-date nature. That excludes any partial and misleading methods of informing, aimed solely at obtaining consent for the activity suggested without the patient’s genuine and reasoned acceptance thereof.

The fundamental characteristics of information proposed by the law recognize the need for communication skills on the part of the healthcare professional towards the patient, so much so that Art. 1 states: “8. The communication time between the doctor and patient constitutes care time. 9. Every public and private healthcare facility shall guarantee the full and accurate implementation of the principles referred to in this law by their own organizational means, ensuring that patients receive the necessary information and that personnel receive adequate training. 10. The initial and continued training of doctors and others in the healthcare profession includes training in matters of relating to and communicating with the patient, pain therapy and palliative care.” Each healthcare professional is required to carry out the active process of communication with the person: the former provides information, and the latter asks for explanations and clarification of that which has been recommended. This relationship of communication is developed throughout the entire care process [144,145,146].

The process of informing the patient through the consent procedure is not simply the correct use of forms or written material that may contain details on the risks of the procedure or its administration; on the contrary, it is a fundamental part of the entire care process and adheres to important principles, ethical and legal, that underlie the care and assistance activity in the Western world. This aspect therefore shows an extraordinary centrality in the relationship between doctors and health workers towards patients.

In the case of nutraceuticals, these needs are not lost, and adequate preventive information must be provided to the patient regarding the scientific evidence that prompts the practitioner to prescribe a nutraceutical, any side effects, and the real expectations of treating the pathology through the administration of nutraceuticals. This preventive therapeutic alliance increases the understanding and availability of the patient by increasing therapeutic adherence. On the medico-legal level, it is also increasingly necessary to consider the increased attention to the safety of treatments. In Italy, even the safety of the patient is a right recognized by law, and the mandatory incentive of all organizational and technical measures is envisaged to manage clinical risk and increase patient and operator safety. Every prescriber of nutraceuticals must be aware of this imperative vision and should therefore pay the utmost attention to the possible effects of administration and any interactions with drugs or other pathologies that the patient may present.

## Figures and Tables

**Table 1:** Common cause of peripheral vertigo/dizziness.

Clinical Manifestation	Pathogenesis	Symptoms	Treatment	References
Benign paroxysmal positional vertigo	Mainly idiopathic, but vestibular neuritis, head trauma, and MD may be related to it. Risk factors include advanced age, ear and dental surgery, vitamin D deficiency, perimenopause, and maybe vascular disorders. Dislocation of the otoconia has a crucial role.	Vertigo, nausea, light headiness.	Vestibular maneuvers, vestibular suppressants.	[7,9,10,11,12,13,14,15,16]
Vestibular neuritis	Uncertain aetiology (e.g., vascular, viral, immunologic, or inflammatory).	Nystagmus, posture alteration, or gait abnormalities.	Vestibular suppressants, steroids, rehabilitation.	[7,9,10]
Ménière’s disease	Endolymphatic hydrops generated by genetic, autoimmune/allergic, vascular, infectious, and mechanic conditions.	Hearing loss (sensorineural, in the low and middle frequencies), vertigo attacks, tinnitus, aural fullness.	Dietary/lifestyle approach, diuretics, betahistine, antiemetic benzodiazepines, anticholinergics, antihistamines, phenothiazines, ondansetron, surgery.	[7,9,10]
Bilateral vestibulopathy	The aetiology often remains unclear. Frequent known causes are ototoxic drugs, bilateral MD, meningitis, and genetic mutations.	Postural imbalance and unsteadiness of gait (both worsen in darkness and on uneven ground); oscillopsia (induced by head or body movement).	It may change on the basis of the aetiology. Rehabilitation, vestibular implants, vestibular stimulation.	[17,18]

MD is characterized by hearing loss (sensorineural, in the low and middle frequencies), vertigo attacks, tinnitus, and aural fullness (Table 1). [9,17,18,19].
